# Non-coding RNA-related antitumor mechanisms of marine-derived agents

**DOI:** 10.3389/fphar.2022.1053556

**Published:** 2022-12-01

**Authors:** Zhixia Zhou, Qianqian Cao, Yujing Diao, Yin Wang, Linhai Long, Shoushi Wang, Peifeng Li

**Affiliations:** ^1^ Institute for Translational Medicine, The Affiliated Hospital of Qingdao University, College of Medicine, Qingdao University, Qingdao, China; ^2^ Qingdao Central Hospital, Central Hospital Affiliated to Qingdao University, Qingdao, China

**Keywords:** marine, antitumor, drug, ncRNA, biomarker

## Abstract

In the last two decades, natural active substances have attracted great attention in developing new antitumor drugs, especially in the marine environment. A series of marine-derived compounds or derivatives with potential antitumor effects have been discovered and developed, but their mechanisms of action are not well understood. Emerging studies have found that several tumor-related signaling pathways and molecules are involved in the antitumor mechanisms of marine-derived agents, including noncoding RNAs (ncRNAs). In this review, we provide an update on the regulation of marine-derived agents associated with ncRNAs on tumor cell proliferation, apoptosis, cell cycle, invasion, migration, drug sensitivity and resistance. Herein, we also describe recent advances in marine food-derived ncRNAs as antitumor agents that modulate cross-species gene expression. A better understanding of the antitumor mechanisms of marine-derived agents mediated, regulated, or sourced by ncRNAs will provide new biomarkers or targets for potential antitumor drugs from preclinical discovery and development to clinical application.

## 1 Introduction

High-throughput sequencing technologies have accelerated the study of biological systems related to the biological activity and function of the human genome, improving our understanding of gene expression and regulation (Dutta et al., 2021). The International Human Genome Consortium revealed that the estimated number of human protein-coding genes has shrunk from 35,000 to 20,500, or less than 3% of the total genome sequence, a surprisingly low number for our species (Liang et al., 2000; Clamp et al., 2007). As a result, there has been an explosion in research into the possible functional roles of the other nearly 98% of the human genome that does not encode proteins (Santosh et al., 2015). In fact, almost 90% of the human genome is transcribed, which means that the RNA pool must contain tens of thousands of noncoding RNA (ncRNA) transcripts with little or no protein-forming capacity (Djebali et al., 2012). Many classes of ncRNAs with very different lengths, structures, and functions have been identified and studied by developing next-generation sequencing techniques over the past two decades (Esteller, 2011; Mehta et al., 2021). Accumulating evidence suggests that they have emerged as important regulators of gene expression by affecting chromatin architecture, DNA/RNA/histone modifications, RNA/protein scaffolds, RNA splicing, stability, and translation (Mehta et al., 2021). Hence, they are not only closely related to various physiological processes, such as cell development, differentiation, and DNA repair but also participate in the pathophysiological processes of various diseases, especially tumors (Dutta et al., 2021).

**FIGURE 1:**
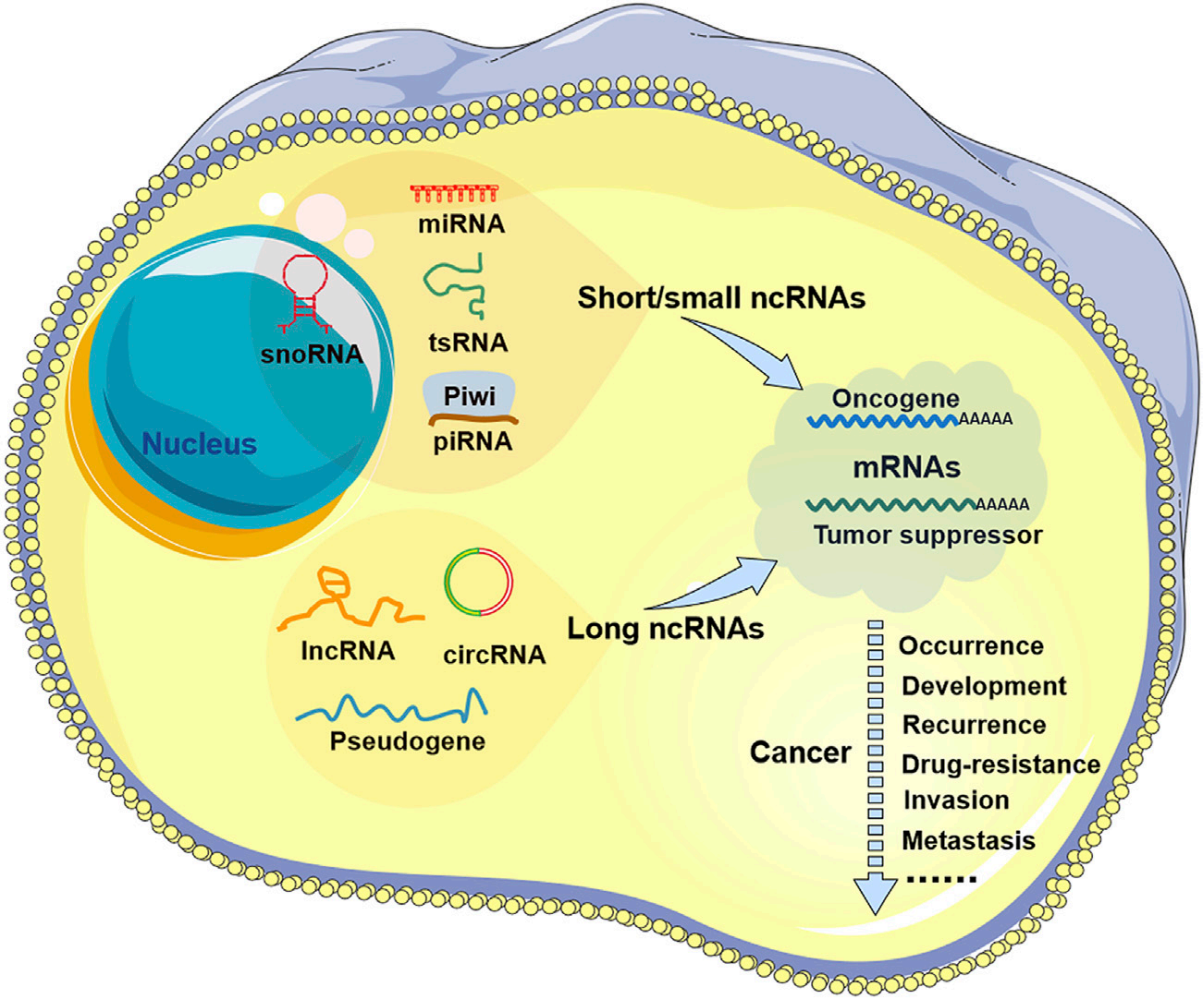
Noncoding RNA in tumors. There are two types of non-coding RNAs (ncRNAs) in tumor cells: short/small ncRNAs and long ncRNAs. Short/small ncRNAs include at least four types: microRNAs (miRNAs), small nucleolar RNAs (snoRNAs), transfer RNA-derived small RNAs (tsRNAs), and piwi-interacting RNAs (piRNAs). Long ncRNAs include common lncRNAs, circular RNAs (circRNAs), and pseudogenes. These ncRNAs are aberrantly expressed in all types of human cancers and are involved in tumor progression, including tumor occurrence, development, recurrence, drug resistance, invasion, and metastasis.

A large body of evidence indicates that ncRNAs are abnormally expressed in all types of human cancers and are associated with patient treatment, prognosis and recurrence (Slack and Chinnaiyan, 2019; Anastasiadou et al., 2018; an and Bu, 2021). Many ncRNAs have been shown to be released from tumor cells into human body fluids, such as blood saliva, urine, and breast milk, which provides strong support for ncRNAs as cancer diagnostic markers or prognostic indicators (Lei et al., 2019; Bautista-Sanchez et al., 2020; Sil et al., 2020). Meanwhile, ncRNAs have also been shown to act as oncogenes or tumor suppressor genes to regulate the occurrence and development of tumors (Figure 1), suggesting their potential as therapeutic targets for antitumor drugs (Arun et al., 2018; Toden et al., 2021; Yan et al., 2022). Moreover, recent studies have shown that some ncRNAs are also implicated in the mechanism of antitumor drugs of natural origin, including marine-derived compounds or derivatives (agents) (Schnekenburger et al., 2014; Liu et al., 2020).

In this review, we first briefly describe the main information known about ncRNAs in tumors and marine-derived antitumor agents. We then focus on the scientific progress made regarding the antitumor mechanisms of various marine-derived agents associated with ncRNAs. In addition, we recapitulated the potential value of marine food-derived ncRNAs as antitumor agents by mediating gene expression across species. This helps to elucidate the antitumor mechanism of marine-derived antitumor agents and reveal the potential value of ncRNAs as biomarkers for marine drug-related tumor treatment, prognosis and recurrence and even as sources of marine antitumor drugs.

## 2 ncRNA in tumors

According to length, ncRNAs can be divided into two categories: short/small ncRNAs and long ncRNAs (lncRNAs). Short/small ncRNAs are transcribed RNA molecules less than 200 nt in length and usually <30 nt. This type of ncRNA includes at least four types: microRNAs (miRNAs), small nucleolar RNAs (snoRNAs), transfer RNA-derived small RNAs (tsRNAs), and piwi-interacting RNAs (piRNAs) (Cech and Steitz, 2014; Slack and Chinnaiyan, 2019). Among them, the most abundant and most investigated short/small ncRNAs are miRNAs, which generally act as negative gene regulators of their target RNAs, especially messenger RNAs (mRNAs), through RNA cleavage, degradation, or translational repression (Vishnoi and Rani, 2017; Slack and Chinnaiyan, 2019). Long ncRNAs are generally longer than 200 nt, and their transcripts consist of multiple exons, 5′ caps and 3′ poly(A) tails but lack open reading frames (ORFs) (Clark and Mattick, 2011). Compared with miRNAs, most lncRNAs have lower sequence conservation among species, but they are implicated in almost all levels of gene expression: epigenetic, transcriptional, and posttranscriptional. They usually interact with other nucleic acid molecules (such as DNA, mRNA, and miRNA) or proteins to act as molecular signals, decoys, guides, or scaffolds (Wang and Chang, 2011; Robinson et al., 2020). In addition, two special cases of lncRNAs have received extensive attention, namely, pseudogenes and circular RNAs (circRNAs) (Groen et al., 2014; Grander and Johnsson, 2016; Wilusz, 2018; Kristensen et al., 2019; Zhou et al., 2020), whose roles and mechanisms remain to be further explored. Dysregulation of both short/small and long ncRNAs can contribute to tumorigenesis and progression (Lekka and Hall, 2018).

### 2.1 miRNAs

miRNAs are by far the most well-characterized ncRNA group in cancer, with more than 70,000 publications in PubMed concerning cancer-related miRNAs. This miRNA dysregulation or genetic changes have been observed in nearly all cancer types studied, and more than 50% of miRNA genes are located in cancer-associated genomic regions or in fragile sites, indicating the important role for miRNAs in the pathogenesis of human cancers (Zhang et al., 2007; Di Leva et al., 2014). According to their effects on tumors, these cancer-associated miRNAs are mainly divided into two subclasses: oncogenic miRNAs (or “tumor-miRs”) and tumor suppressor miRNAs (Yan et al., 2022). Examples of well-characterized oncogenic miRNAs include miR-21, miR-141, miR-155, and miR-215, which are widely overexpressed in a variety of systemic tumors, including gastrointestinal, respiratory, hematological, and reproductive system tumors (Bayraktar and Van Roosbroeck, 2018; Vychytilova-Faltejskova and Slaby, 2019; Luo et al., 2022; Yan et al., 2022). Conversely, the let-7 family, miR-34a, miR-145, and miR-29 targets are regarded as tumor suppressors and can inhibit tumor formation, proliferation, migration, spread, and angiogenesis by targeting many oncogenic transcription factors, including E2F1, K-RAS and c-MYC (Yang et al., 2015; Alizadeh et al., 2019; Zhang et al., 2019; Xu et al., 2020). Interestingly, several miRNAs appear to possess dual functionality, acting as both tumor suppressors and oncogenes. Interestingly, some miRNAs seem to have a double-edged sword-like effect on tumor progression, playing diametrically opposite promotion or inhibition effects on tumors at different stages, such as miR-200c (Korpal et al., 2011; Pecot et al., 2013; Toiyama et al., 2014; Yan et al., 2022). In agreement with their oncogenic or tumor-suppressive roles, miRNA-targeting cancer therapeutic strategies include miRNA mimics, small-molecule antagonists/drugs, or miRNA inhibitors based on oligonucleotide, sponge, or CRISPR/Cas9 genome editing (Regazzi, 2018; He et al., 2020; Yan et al., 2022). The therapeutic strategies for miRNA delivery mainly rely on liposomes, cationic polymers, peptide-based transmembrane structures, adeno-associated viral vectors, and exosomes (Arun et al., 2018; Liu et al., 2020; Sil et al., 2020).

### 2.2 lncRNAs

To date, approximately 60,000 aberrantly expressed lncRNAs have been identified from human tumor tissues and cancer cell lines, and although the functions of most RNAs in tumors are not well defined, there are approximately 30,000 publications about cancer-related lncRNAs on PubMed (Iyer et al., 2015). Similar to miRNAs, cancer-related lncRNAs also act as oncogenes or suppressors to regulate cancer initiation, angiogenesis, metastasis, and drug resistance. Among them, the more well-studied oncogenic lncRNAs are mainly GHET1, ANRIL, MALAT1, PCAT-1, HOTAIR, HOTTIP, and PCA3, which are often overexpressed in cancer and are associated with poor patient outcomes (Clark and Mattick, 2011; Regazzi, 2018). In contrast, GAS5, MEG3, LincRNA-p21, NCRNACCND1, and CASC15-S, which are cancer suppressors, are generally underexpressed in cancer and are related to good patient prognosis (an and Bu, 2021; Yan et al., 2022; Tang et al., 2017). The functional mechanism of these lncRNAs in tumor cells involves a variety of cell signaling pathways related to cellular processes, such as p53, nuclear transcription factor-κB (NF-κB), phosphatidyl inositol 3-kinase (PI3K)/protein kinase B (AKT), extracellular regulated protein kinases (ERK)/mitogen-activated protein kinase (MAPK), wingless-type MMTV integration site family (Wnt)/β-chain protein (β-catenin), and hypoxia-inducible factor-1α (HIF1α) (Peng et al., 2017). In addition, the competing endogenous RNA (ceRNA) regulatory network of lncRNA‒miRNA-mRNA is an important molecular mechanism for ncRNA functions in tumor pathogenesis (Lei et al., 2019). Emerging therapeutic strategies targeting lncRNAs mainly focus on the clearance of oncogenic lncRNAs mediated by RNA interference (RNAi), morpholino-based antisense oligonucleotides (MO-ASOs), natural antisense RNAs (NATs), CRISPR‒Cas9 genome editing, *etc.* (Schnekenburger et al., 2014; Slack and Chinnaiyan, 2019).

### 2.3 Other ncRNAs

The success of miRNA-based cancer research has promoted not only the research progress of lncRNAs but also research on other ncRNA families and identified them as potential therapeutic targets for cancer, such as circRNAs, tsRNAs, piRNAs, and snoRNAs. Compared with the first two ncRNAs, there are relatively few studies on these ncRNAs. For example, there are fewer than 5,000 literature reports on tumor-related circRNAs on PubMed. The expression profiles of circRNAs in human cancers are diverse, depending on the type of cancer (Li et al., 2021). That is, each type of tumor has only its own unique abnormally expressed RNA, and the expression of a given circRNA is significantly different in different cancer types. Therefore, the tissue and cancer specificity of circRNA expression profiles makes them potentially reliable prognostic markers for cancer diagnosis and therapy. At present, well-characterized tumor-related circRNAs are mainly from breast, lung, liver, and gastric cancers. These circRNAs have been reported to be involved in cancer development as oncogenes or tumor suppressor genes, ranging from tumor growth and metastasis to drug resistance, such as circAGFG1, CircDnmt1, circHER2 circTADA2A-E6, and Circ0025202 (Kristensen et al., 2018; He et al., 2021; Zhou et al., 2021). tsRNAs are the second most abundant member of the sncRNA family after miRNAs (Lee et al., 2009). They are often highly expressed in cancers under stress and correlate with the clinical stage of cancer (Fu et al., 2009; Gebetsberger and Polacek, 2013). Moreover, tsRNA expression levels are also elevated in the serum and urine of cancer patients, suggesting their potential as diagnostic markers for cancer (Lakings et al., 1977; Gehrke et al., 1979). piRNAs were originally thought to be mainly expressed in the reproductive system but not in cancer (Siddiqi and Matushansky, 2012). Recently, increasing evidence has shown that piRNAs are not only abnormally expressed in cancer but also related to the mechanism of tumor occurrence and development. For example, piRNA-823, piRNA-54265A, and piR-651 are highly expressed in tumors and play a role in promoting cancer development (Feng et al., 2020; Mai et al., 2020). Similarly, the functions of snoRNAs have been considered for many years to be those of housekeeping genes; however, there is increasing evidence that high expression of certain snoRNAs also has oncogenic effects (Thorenoor and Slaby, 2015), such as snoRNA U50 and SNORA42 (Tanaka et al., 2000; Mei et al., 2012). However, despite the important roles of these ncRNAs in cancer, their functional and mechanistic studies in tumorigenesis are still at an early stage and require further investigation.

## 3 Marine-derived antitumor agents

As described above, the emergence of tumor-related ncRNAs provides a new alternative for cancer diagnosis and therapy. However, compared with many other potential targets (oncogenes, mutations, gene fusion, *etc.*), the advantages of targeted ncRNA therapy in cancer are still unclear, and their effective application in clinical cancer intervention remains challenging and is limited by many obstacles, such as off-target effects, toxicity of the delivery system, and synthetic ncRNA immunogenicity (Peng et al., 2017). At present, chemotherapy is one of the most effective methods for the treatment of cancer, especially for patients with advanced tumors or with a tendency to spread throughout the body. However, it is a systemic treatment that allows them to destroy or eliminate tumor cells while also causing damage to normal cells, bringing adverse reactions to patients. In addition, tumor cells have a certain resistance to the effects of chemotherapeutic drugs through self-regulation, which significantly reduces the efficacy of chemotherapeutic drugs (Perez-Herrero and Fernandez-Medarde, 2015). Therefore, these limitations limit the clinical application of chemotherapeutic drugs and are further incentives to find other effective treatments with fewer side effects.

**FIGURE 2:**
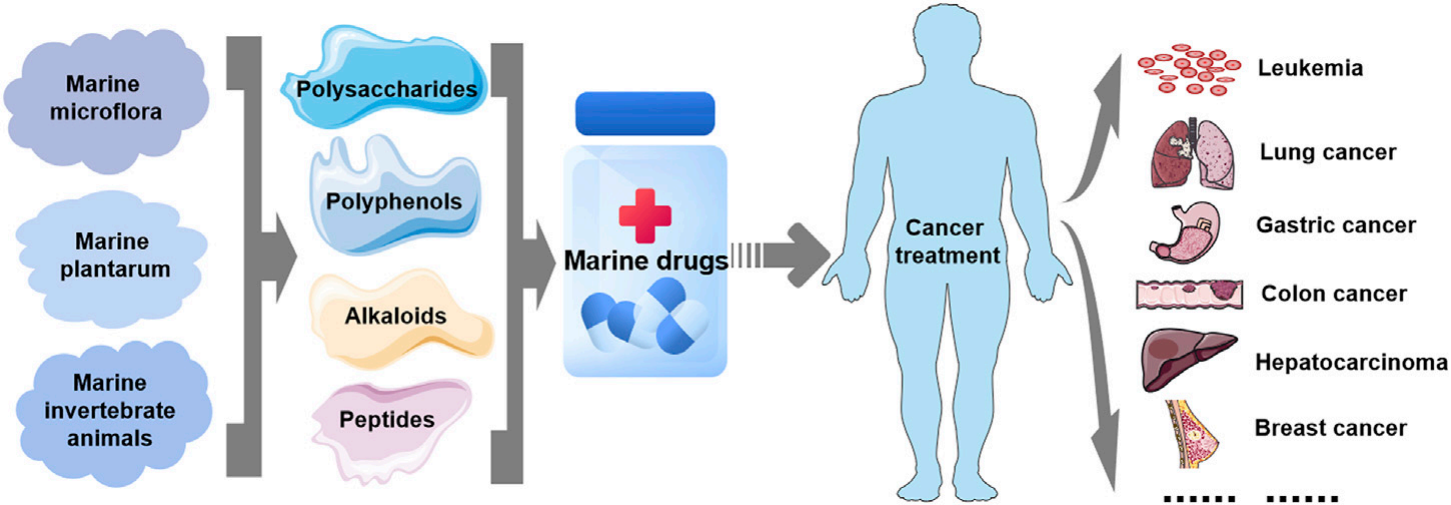
Marine-derived antitumor drugs. Antitumor substances of marine natural origin include polysaccharides, polyphenols, alkaloids, and peptides extracted from marine organisms, including microflora, plants and invertebrates. At present, several marine-derived antitumor substances have been used as drugs for the treatment of clinical human tumors, including leukemia and lung, gastric, colon, liver, breast, and liver cancers.

Over the past 20 years, emerging evidence has shown that natural products, including marine-derived compounds or derivatives, represent an available source of antitumor drugs, new drugs and chemical entities, whose biologically active ingredients exhibit potent cytotoxic effects on tumor cells with few side effects (Newman et al., 2003; Wu et al., 2021). The antitumor effects of these marine-derived substances have been or are being demonstrated *in vitro*, *in vivo* (animal models), and in preclinical and clinical trials (Newman and Cragg, 2012; Newman and Cragg, 2014). Many have been approved as antitumor drugs for clinical cancer treatment, such as cytarabine, eribulin mesylate, and trabectidine (Khalifa et al., 2019). As shown in Figure 2 these marine-derived agents derived from marine plants and microbes, the Caribbean sponges (*Cryptotethya crypta*), were the first to be studied with tumor cytotoxicity, followed by those derived from many other organisms, ranging from marine microflora (bacteria, actinomycetes, cyanobacteria, and fungi) and plantarum (microalgae and macroalgae) to invertebrate animals (e.g., sponges, soft corals, nudibranchs, and tunicates) (Khalifa et al., 2019). However, the antitumor mechanisms of these marine-derived agents remain to be further studied. With the advancement of science and technology and extensive research on the ocean, such as deep-sea mining, extraction and separation, molecular modification, genetic engineering, and organic synthesis (Wu et al., 2021), an increasing number of organisms and their metabolites have been used in the study of marine natural antitumor agents. However, the antitumor mechanism, clinical safety and treatment strategy of the active ingredients of marine-derived agents still need to be further studied. Evidence suggests that their toxic effects on tumor cells are achieved by targeting cancer-related molecules expressed by cancer cells, such as macromolecules in signal transduction pathways (Newman et al., 2003; Wu et al., 2021). Moreover, recent studies have shown that several small molecules are also involved in the antitumor mechanism of marine-derived substances, including ncRNAs.

## 4 ncRNAs as oncogenes or tumor suppressors involved in the antitumor mechanism of marine-derived agents

**FIGURE 3:**
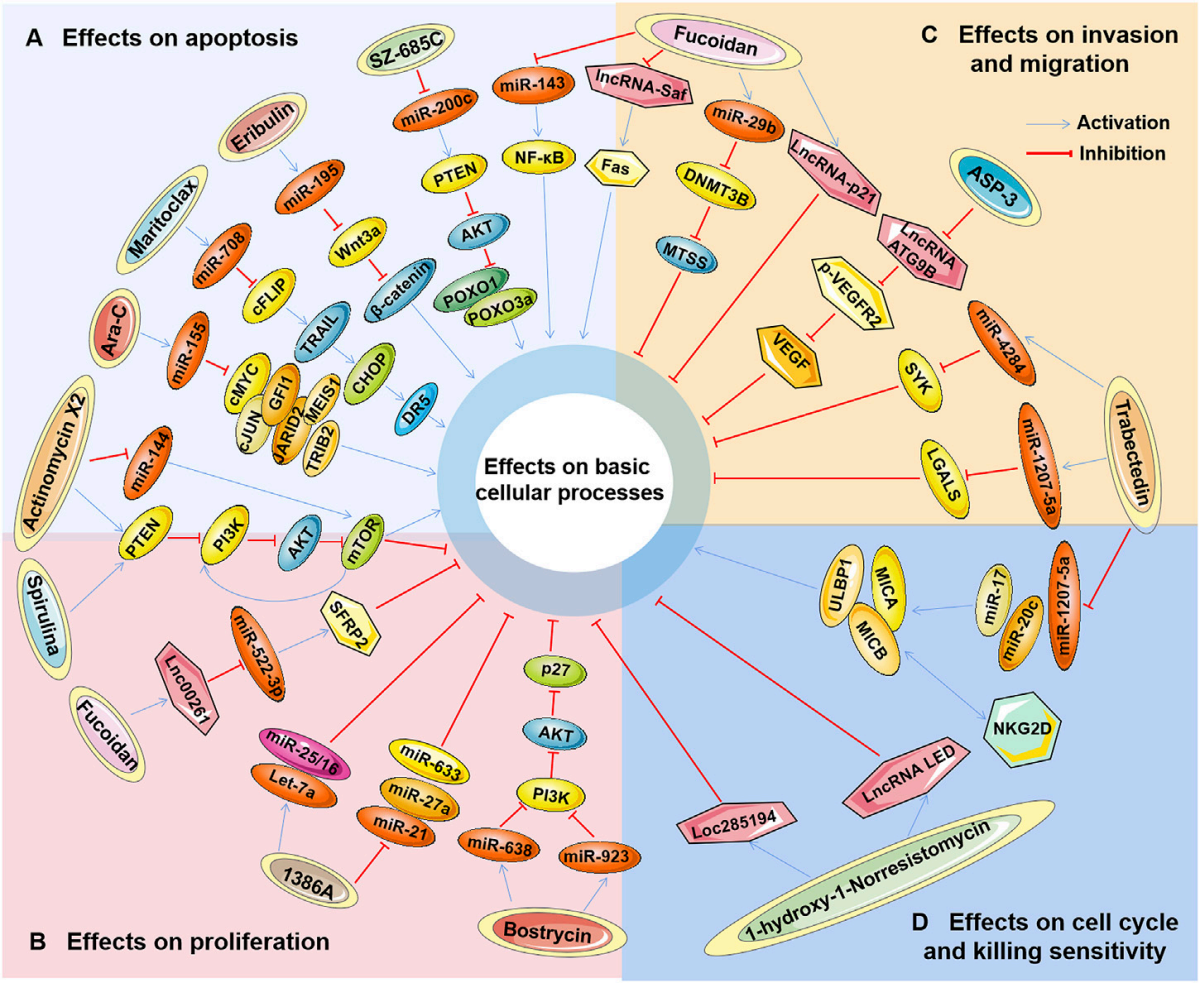
The antitumor effects of marine-derived antitumor agents mediated by non-coding RNAs in basic cellular processes. Marine-derived antitumor agents (SZ-685C, eribulin, maritoclax, trabectedin, spirulina, bostrycin, cytarabine, fucoidan, 1386A, actinomycin X2,1-hydroxy-1-norresistomycin, and ASP-3) can induce tumor cell apoptosis **(A)**, inhibit cell proliferation **(B)**, invasion, and metastasis **(C)**, inhibit cell cycle arrest, and enhance tumor cell-killing sensitivity to immune cells **(D)** by upregulating oncogenic noncoding RNA (ncRNA), or downregulate tumor suppressor ncRNAs.

**FIGURE 4:**
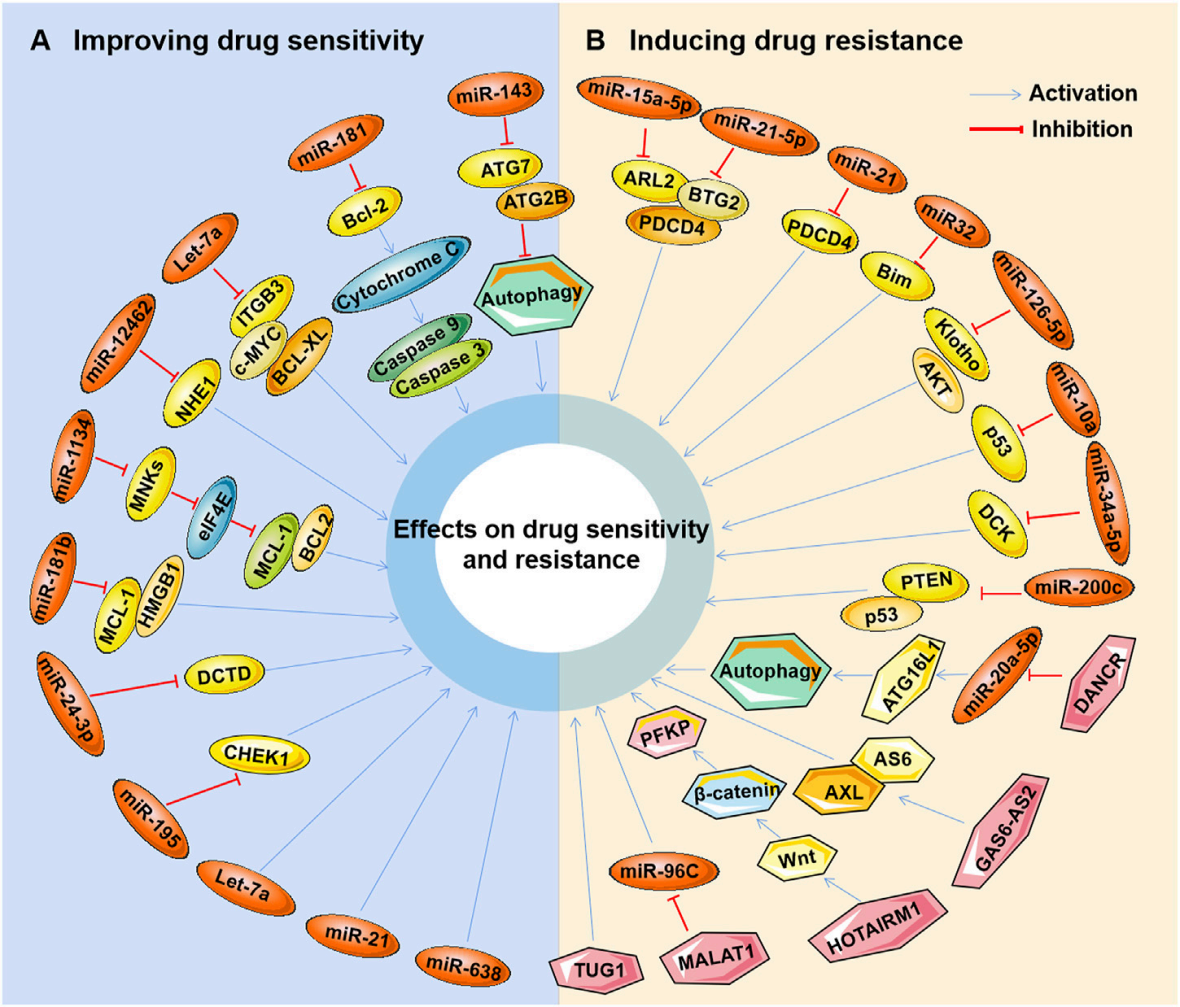
Marine-derived antitumor agent-related non-coding RNAs in tumor cell drug sensitivity and resistance. Non-coding RNAs (ncRNAs) regulated by marine-derived antitumor agents (cytarabine or trabectedin) can be used as tumor suppressors to induce the drug sensitivity of tumor cells **(A)** or as oncogenes to induce cellular drug resistance **(B)**.

Studies have found that marine-derived agents exert antitumor activity by interfering with basic cellular processes of tumor cells, such as proliferation, apoptosis, autophagy and the cell cycle. Mechanistically, it was found that several marine substances could affect the expression levels of ncRNAs (mainly miRNAs and lncRNAs) in tumor cells and then affect their functions so that the antitumor functions of these marine-derived substances depend at least to some extent on the ncRNAs they affect, including SZ-685C, eribulin, maritoclax, trabectedin, spirulina, bostrycin, cytarabine, fucoidan, 1386A, actinomycin X2, 1-hydroxy-1-norresistomycin, and ASP-3 Figure 3 and (Table 1). Furthermore, the sensitivity or tolerance of tumor cells to these marine-derived agents (cytarabine and trabectedin) can also be affected by these marine-agent-related ncRNAs, as shown in Figure 4 and Table 2. The data suggest that marine-derived agent-related ncRNAs can act as oncogenes or tumor suppressor genes to mediate or regulate the antitumor activity of marine-derived agents.

**TABLE 1:** Marine-derived agents associated with ncRNAs in tumor fundamental cellular processes.

Marine-derived agents (source)	Related ncRNAs	ncRNA expression regulated by agents	ncRNA roles in tumor	Related cellular processes	Related genes	Tumor types	References
miRNA-related marine-derived agents							
SZ-685C (Angrove endophytic fungus)	miR-200c	Down-regulated	Oncogene	Inhibition of cell proliferation, induction of apoptosis	PTEN, p-AKT, FOXO1, FOXO3a	Pituitary tumor	Xie et al., (2010); Chen et al., (2013a); Liao et al., (2013)
Eribulin (*Halichondria okadai*)	miR-195	Up-regulated	Suppressor	Induction of apoptosis	Wnt3a, β-catenin	Breast cancer	Green et al., (2013); Furuya et al., (2016); Yu et al., (2018a)
Maritoclax (*Streptomyces*)	miR-708	Up-regulated	Suppressor	Induction of apoptosis	cFLIP, TRAIL, CHOP, DR5	Renal carcinoma	Jeon et al., (2018); Sun et al., (2019)
Trabectedin (*Ecteinascidia turbinata*)	miR-4284, miR-1207-5, miR-1225-5p	Up-regulated	Suppressor	Induction of apoptosis, inhibition of cell migration and motility	SYK, LGALS1	Biliary tract cancer	Peraldo Neia et al. (2016)
	pri-miR-17-92, miR-17, miR-20a	Up-regulated	Suppressor	Induction of apoptosis, cell cycle arrest, cellular stress, and DNA damage	IRF4, IKZF1, MICA, MICB, ULBP1, NKG2D	Multiple myeloma	Cuce et al. (2019)
Spirulina (*Cyanobacteria*)	miR-34a, miR-125b	Up-regulated	Suppressor	Inhibition of cell proliferation	PI3K, AKT, mTOR	Glioma	Arab et al., (2021); Braune et al., (2021)
Bostrycin (*Alternaria eichhorniae*)	miR-638, miR-923	Up-regulated	Suppressor	Inhibition of cell proliferation	PT3K, AKT, p27	Lung cancer	Chen et al., (2011); Chen et al., (2012a)
Cytarabine (*Cryptotethia crypta*)	miR-155	Up-regulated	Suppressor	Inhibition of cell proliferation, induction of cell differentiation and apoptosis	cMYC, cJUN, GFI1, JARID2, MEIS1, TRIB2	Acute myeloid leukemia	Palma et al. (2014)
Fucoidan (Brown seaweed)	miR-29, miR-143	Up-regulated	Suppressor	Inhibition of cell survival, proliferation, invasive, and EMT process	DNMT3B, MTSS1, NF-κB pathway	Hepatocellular carcinoma	Yan et al., (2015); Zheng et al., (2018); El-Far et al., (2021)
1386A (Mangrove fungus)	let-7a, miR-15/16	Up-regulated	Suppressor	ND	ND	Breast cancer	Tang et al. (2012)
	miR-21, miR-27a, miR-633	Down-regulated	Oncogene	ND	ND	Breast cancer	Tang et al. (2012)
Actinomycin X2 (*Streptomyces*)	miR-144	Up-regulated	Oncogene	Inhibition of cell proliferation, induction of apoptosis	PTEN, PI3K, AKT, mTOR	Prostate cancer	Liu et al. (2016)
lncRNA-related marine-derived agentsH							
Fucoidan (Brown seaweed)	LINC00261	Up-regualted	Suppressor	Inhibition of cell proliferation and invasion	miR-522-3p, SFRP2	Hepatocellular carcinoma	Ma et al. (2021)
	lncRNA-p21	Up-regualted	Suppressor	Inhibition of cell invasion and metastasis	ND	Hepatocellular carcinoma	Yan et al. (2019)
	lncRNA-Saf	Down-regualted	Oncogene	Induction of apoptosis	Fas	Hepatocellular carcinoma	Yan et al., (2005); Yan et al., (2019)
1-hydroxy-1-Norresistomycin (*Streptomyces*)	LED, LOC285194	Up-regualted	Suppressor	Inhibition of cell proliferation, invasion, and metastasis, induction of apoptosis and cell cycle arrest	p53, p21	Lung cancer	Ramalingam et al. (2018)
ASP-3 (*Arca subcrenata*)	ATG9B	Down-regualted	Oncogene	Inhibition of invasion and metastasis	VEGFR2	Hepatocellular carcinoma	Guo et al. (2019)

ND, not determined.

**TABLE 2:** Marine-derived agents associated with ncRNAs in tumor drug sensitivity and resistance.

Aghents (source)	Related ncRNAs	ncRNA expression regulated by agents	ncRNA roles in tumor	Related cellular processes	Related genes	Tumor types	References
miRNA-related marine-derived agents							
Cytarabine (*Cryptotethia crypta*)	miR-143	Down-regulated	Suppressor	Promotion or maintenance of cellular drug sensitivity	ATG7, ATG2B	Acute myeloid leukemia	Zhang et al. (2020c)
	miR-181a	Down-regulated	Suppressor	Promotion or maintenance of cellular drug sensitivity	Bcl-2, Caspase 9/3, Cytochrome C	Acute myeloid leukemia	Bai et al. (2012)
	miR-12462	ND	Suppressor	Promotion or maintenance of cellular drug sensitivity	NHE1	Acute myeloid leukemia	Jia et al. (2020)
	let-7a	ND	Suppressor	Promotion or maintenance of cellular drug sensitivity	SDF-1α, CXCR4, YY1, ITGB3, c-MYC, BCL-XL	Acute myeloid leukemia	Chen et al. (2013b)
	miR-29a, miR-30c, miR-625-3p	ND	Suppressor	Promotion or maintenance of cellular drug sensitivity	ND	Acute myeloid leukemia	Russ et al., (2011); Jiang et al., (2018)
	miR-134	Down-regulated	Suppressor	Promotion or maintenance of cellular drug sensitivity	eIF4E, MNKs, MCL-1, BCL2	Acute myeloid leukemia	Chen et al. (2018a)
	miR-181b	Down-regulated	Suppressor	Promotion or maintenance of cellular drug sensitivity	Mcl-1, HMGB1	Acute myeloid leukemia	Lu et al. (2014)
	miR-24-3p	Down-regulated	Suppressor	Promotion or maintenance of cellular drug sensitivity, inhibition of autophagy	DCTD	Acute myeloid leukemia	Bhise et al. (2019)
	miR-15a-5p, miR-21-5p	UP-regulated	Oncogene	Induction of cellular drug resistance	PDCD4, ARL2, BTG2	Acute myeloid leukemia	Vandewalle et al. (2021)
	miR-21	UP-regulated	Oncogene	Induction of cellular drug resistance	PDCD4	Acute myeloid leukemia	Vandewalle et al. (2021)
	miR-32	UP-regulated	Oncogene	Induction of cellular drug resistance	Bim	Acute myeloid leukemia	Vandewalle et al. (2021)
	miR-126-5p	UP-regulated	Oncogene	Induction of cellular drug resistance	*Klotho*, Akt	Acute myeloid leukemia	Shibayama et al. (2015)
	miR-10a	UP-regulated	Oncogene	Induction of cellular drug resistance	p53	Acute myeloid leukemia	Vu et al. (2021)
	miR-34a-5p	UP-regulated	Oncogene	Induction of cellular drug resistance	DCK	Acute myeloid leukemia	Bhise et al. (2019)
	miR-200	UP-regulated	Oncogene	Induction of cellular drug resistance	PTEN, p53	Colon cancer	Chen et al. (2014)
Trabectedin (*Ecteinascidia turbinata*)	miR-130a, miR-19a, miR-125a-5p...	Up-regulated	Oncogene	Induction of cellular drug resistance	FUS-CHOP fusion gene	Myxoid liposarcoma	Grosso et al., (2007); Forni et al., (2009); Uboldi et al., (2012)
	miR-195	ND	Suppressor	Promotion or maintenance of cellular drug sensitivity	CHEK1	Lung cancer	Yu et al. (2018b)
lncRNA-related marine-derived agents							
Cytarabine (*Cryptotethia crypta*)	DANCR	Up-regualted	Oncogene	Induction of cellular drug resistance and autophagy	miR-20a-5p, ATG16L1	Acute myeloid leukemia	Zhang et al. (2021b)
	GAS6-AS2	Up-regualted	Oncogene	Induction of cellular drug resistance	AS6, AXL	Acute myeloid leukemia	Bester et al. (2018)
	HOTAIRM1	ND	Oncogene	Induction of cellular drug resistance, glucose consumption, and lactate production	Wnt, β-catenin, PFKP	Acute myeloid leukemia	Chen et al. (2020)
	MALAT1	ND	Oncogene	Induction of cellular drug resistance	miR-96C	Acute myeloid leukemia	Goyal et al. (2021)
	TUG1	Up-regualted	Oncogene	Induction of cellular drug resistance	ND	Acute myeloid leukemia	Luo et al. (2018)

ND, not determined.

### 4.1 Regulation of basic cellular processes in tumor cells (apoptosis, proliferation, cell cycle, invasion, and migration) by ncRNA-related marine-derived drugs

#### 4.1.1 miRNA-related marine-derived agents

SZ-685C is a marine anthraquinone isolated from a mangrove endophytic fungus, and its anticancer effects have been demonstrated in various tumors, including breast, prostate, liver, and pituitary cancers and glioma (Xie et al., 2010; Chen et al., 2013a). SZ-685C inhibited the proliferation ability of tumor cells *in vitro* and xenograft tumor growth *in vivo* (Xie et al., 2010; Chen et al., 2013a). In addition, SZ-685C has a direct proapoptotic effect by downregulating the phosphorylation of AKT (p-AKT) and its downstream factors forkhead box protein O1 (FOXO1) and FOXO3a (Xie et al., 2010). Regarding pituitary tumor cells, the apoptotic capacity induced by Z-685C exhibited a microRNA-200c (miR-200c) dependency (Chen et al., 2013a). Treatment with SZ-685C significantly downregulated the expression level of miR-200c in tumor cells, while overexpression of miR-200c significantly attenuated SZ-685C-induced apoptosis (Chen et al., 2013a). MicroRNA-200c has been reported to act as an oncogene to inhibit the apoptosis of pituitary adenoma cells by decreasing phosphatase tensin homolog (PTEN) expression but upregulating the expression of p-AKT (Liao et al., 2013). These results suggest that SZ-685C exerts antitumor effects by targeting the miR-200c/PTEN/Akt/FOXO pathway to induce apoptosis.

Eribulin (eribulin mesylate), a synthetic analog of a natural polyether macrolide derived from marine sponges, is a tubulin-interacting cytotoxic agent that was approved for the treatment of metastatic breast cancer and soft tissue sarcoma (STS) (Seetharam et al., 2018). The antitumor activity of eribulin is mainly manifested by downregulating Rho to inhibit cell migration and by promoting cell apoptosis through upregulating p53 and BAX (Wiemer et al., 2017). Moreover, studies have shown that miRNAs are also involved in the antitumor mechanism of eribulin. Furuya reported that the expression profiles of miRNAs in different types of triple-negative breast cancer (TNBC) cells treated with eribulin were significantly different, including miR-195, which has been reported to significantly impact oncogenicity in various cancers (Furuya et al., 2016; Yu et al., 2018a). Compared with the basal-like TNBC cells of HCC1143, the expression level of miR-195 was significantly decreased compared to the nonbasal-like TNBC cells of MDA-MB 231. Correspondingly, the expression of Wnt3a, a target of miR-195, was significantly lower in MDA-MB-231 cells treated with eribulin (Furuya et al., 2016). Since the inhibition of Wnt3a can lead to the inactivation of the Wnt/β-catenin signaling pathway and induce cancer cell apoptosis, the apoptosis level of MDA-MB-231 cells after eribulin treatment is significantly higher than that of HCC1143 cells (Green et al., 2013; Furuya et al., 2016). These results confirm the role of miRNAs in the antitumor effect of eribulin and may explain the different susceptibilities to eribulin in patients with different breast cancer types.

Maritoclax, a natural compound isolated from marine *Streptomyces*, is a B-cell lymphoma-2 (BCL-2) family inhibitor that induces proteasomal degradation of myeloid cell leukemia-1 (MCL-1) (Haste et al., 2011; Doi et al., 2012). The antitumor effect of maritoclax by inducing apoptosis has been demonstrated in tumors (Pandey et al., 2013; Doi et al., 2014; Varadarajan et al., 2015; Jeon et al., 2018). In addition, maritoclax was recently shown to enhance TRAIL-induced apoptosis in tumor cells. Jeon found that combined treatment with maritoclax and TRAIL markedly induced apoptosis in different types of tumor cells, including kidney, lung, and liver cancer cells (Jeon et al., 2018). C/EBP homologous protein (CHOP)-mediated upregulation of death receptor 5 (DR5) was then proven to be involved in the enhancement of tumor necrosis factor-related apoptosis-inducing ligand (TRAIL)-induced apoptosis by maritoclax (Jeon et al., 2018). Meanwhile, the expression of miR-708, which is considered an oncogene or a tumor suppressor gene in different tumors, was significantly increased by maritoclax (Jeon et al., 2018; Sun et al., 2019). Furthermore, it was found that miR-708 could mediate the downregulation of cellular FLICE-inhibitory protein (cFLIP), which played a negative role in the enhancement of TRAIL-induced apoptosis by maritoclax (Jeon et al., 2018). This suggests that miR-708 acts as a tumor suppressor gene involved in the apoptosis induced by maritoclax combined with TRAIL.

Trabectedin (Ecteinascidin-743 or NSC 684766) is a tetrahydroisoquinoline alkaloid isolated from the marine tunicate *Ecteinascidia Turbinata* that has a potent antitumor effect by regulating various transcription factors involved in cell proliferation and the cell cycle (Trabectedin, 2006). Trabectedin has been approved for the clinical treatment of cancer, primarily ovarian cancer and soft tissue sarcoma (Krasner et al., 2012; Di Giandomenico et al., 2014). Trabectedin was also found to have antitumor activity against other types of tumors, including biliary tract cancer (BTC) and multiple myeloma (MM) (Peraldo Neia et al., 2016; Cuce et al., 2019). In trabectedin-treated BTC cells, the expression of genes involved in tissue development and apoptotic processes was activated, whereas genes involved in cell migration and motility were inhibited, including spleen tyrosine kinase (SYK) and lectin galactoside-binding soluble 1 (LGALS1). Further research showed that silencing SYK or LGALS1 significantly inhibited the migration of BTC cells (Peraldo Neia et al., 2016). miRNA expression profiling of trabectedin-treated BTC cells revealed that 24 microRNAs were deregulated by trabectedin, while 5 microRNAs were perturbed. Interestingly, SYK is a putative target of all upregulated microRNAs, including miR-4284, while LGALS1 is a predicted target of miR-1207-5p and miR-1225-5p (Peraldo Neia et al., 2016). All three miRNAs have been reported to function as tumor suppressor genes in cancer (Aydogdu et al., 2012; Yang et al., 2014), which suggests further investigation into the interaction of these miRNAs with their targets SYK and LGALS1. Regarding multiple myeloma (MM) cells, trabectedin also exerts potent antitumor activity by inducing apoptosis, cell cycle arrest, cellular stress, and DNA damage *in vitro* and in 3D models (Baruchel et al., 2012). Moreover, trabectedin inhibited the expression of interferon regulatory factor 4 (IRF4), IKAROS family zinc finger 1 (IKZF1) and the miR-17 family (pri-miR-17-92, miR-17, and miR-20a) in MM cells, resulting in the upregulation of MICA, MICB, and ULBP1, ligands of natural killer (NK) cell activating receptor NKG2D, which ultimately lead to the activation and degranulation of NK cells (Cuce et al., 2019). These results suggest that trabectedin treatment not only induces tumor cell suppression but also enhances their lysis susceptibility to immune cells.

Spirulina (Spi), a nutritionally valuable dietary supplement isolated from blue‒green algae (*Cyanobacteria*), has long been reported to have antitumor properties (ElFar et al., 2022). Further study found that Spi exhibits significant anti-proliferative effects on glioma cells in both *in vitro* and *in vivo* models by decreasing the expression of PI3K/AKT/mammalian target of rapamycin (mTOR) and increasing the expression of miR-34a and miR-125b (Arab et al., 2021; Braune et al., 2021). Both miR-34a and miR-125a-5p were reported to have suppressive effects in cancer *via* the PI3K/AKT/mTOR pathway (Li et al., 2018; Zhang et al., 2020a). Reports suggest that Spi exerts antitumor effects by targeting the miR-34a/PI3K/AKT/mTOR or miR-125b/PI3K/AKT/mTOR pathway to inhibit cell proliferation.

Bostrycin is a classical compound isolated from marine fungi, and its antitumor activity has been demonstrated in prostate, gastric, and lung cancers (Chen et al., 2011; Chen et al., 2012a; Jie et al., 2020). It can significantly inhibit the proliferation of tumor cells, promote apoptosis, and cause cell cycle arrest in the G0/G1 phase (Chen et al., 2011; Chen et al., 2012a). The expression levels of microRNA-638 and microRNA-923 were significantly upregulated in bostrycin-treated lung adenocarcinoma cells. Furthermore, the downregulation of AKT and the upregulation of the cell cycle protein p27 were shown in bostrycin-treated cells (Chen et al., 2011). microRNA-638 has been reported to function as a tumor suppressor gene in tumor cells to regulate cell proliferation, apoptosis, metastasis and autophagy (Wang et al., 2017a; Zhang et al., 2021a; Xu et al., 2021). However, the role of microRNA-923 in tumors and its mechanism still need further study.

Cytarabine (Ara-C), a nucleoside analog derived from sponge, is the first marine-derived drug approved for the treatment of human leukemia and lymphomatous meningitis by disrupting DNA synthesis to induce cell apoptosis (Burnett et al., 2011). Palma found that Ara-C significantly increased the expression level of miR-155 in acute myeloid leukemia (AML) cells, and inhibition of miR-155 made cells resistant to cytarabine-induced apoptosis and inhibited the ability of cells to differentiate (Palma et al., 2014). Ectopic expression of miR-155 in AML cells resulted in enhanced cell differentiation capacity, increased apoptosis, and decreased cell growth and clonogenic capacity, suggesting that miR-155 has tumor-suppressive effects. It was further confirmed that multiple apoptosis- and differentiation-related transcriptional regulators were target genes of miR-155, including c-MYC, c-JUN, Jumonji (JARID2), myeloid ecotropic viral integration site 1 (MEIS1), growth factor independent 1 transcriptional repressor (GFI1) and tribbles pseudokinase 2 (TRIB2) (Palma et al., 2014). These results suggest that Ara-C could induce apoptosis and differentiation of AML cells by upregulating the tumor suppressor miR-155.

Fucoidan is a sulfated polysaccharide isolated from brown seaweed and has various pharmacological effects, including antitumor effects (Wu et al., 2016; Deepika et al., 2019). It is able to induce apoptosis of various tumor cells *in vitro* by regulating many signaling pathways, such as caspase family proteins, transforming growth factor (TGF) receptor, Smad 4 protein, and secreted frizzled-related protein 2 (SFRP2) (Senthilkumar and Kim, 2014; Ma et al., 2021). A total of 53 miRNAs were affected by fucoidan in a 24-hour test study of healthy volunteers ingesting a single dose of 1 g of fucoidan for 24 h (Gueven et al., 2020). These miRNAs affected 29 different pathways and processes, including cancer-related pathways, consistent with most previous results obtained in the laboratory. This study shows that even a single dose of a fucoidan drug has the potential to affect the expression of genes involved in basic cellular processes (Gueven et al., 2020). Analysis of the microRNA expression profiles of fucoidan-treated HCC cells revealed that the expression level of miR-29b was markedly upregulated by fucoidan. miR-29b was subsequently shown to act as a tumor suppressor gene to inhibit the EMT process by targeting the DNA methyltransferase (DNMT) 3B-metastasis suppressor (MTSS) 1 axis, ultimately leading to a reduction in cell-invasive activity (Yan et al., 2015). Furthermore, in an *in vivo* model of hepatocellular carcinoma (HCC), fucoidan significantly improved survival, inhibited cellular proliferation, and decreased serum AFP expression levels in HCC rats. Additionally, fucoidan reduced the upregulation of miR-143 and NF-κB expression levels induced by HCC (El-Far et al., 2021). The miR-143/NF-κB pathway was proven to be an important component involved in tumor progression and invasion (Zheng et al., 2018). This suggests that fucoidan may inhibit the metastasis of liver cancer by inhibiting the miR-143-mediated signaling pathway but promoting the miR-29-mediated signaling pathway.

The marine fungal metabolite 1386A, a small-molecule compound derived from a mangrove fungus, was reported to significantly inhibit MCF-7 cell proliferation in a time- and dose-dependent manner (Tang et al., 2012). Analysis of miRNA expression profiles induced by 1386A revealed that several deregulated miRNAs may play complex roles in 1386A-induced breast cancer cytotoxicity, including the unregulated tumor suppressor miRNAs (let-7a and miR-15/16) and the downregulated oncogenic miRNAs (miR-21, miR-27a and miR-633) (Tang et al., 2012). This suggests that 1386A may be an effective antitumor drug, and its molecular mechanism may be related to miRNAs, which needs further study.

Marine-derived *Streptomyces* MS449 produces high yields of the polypeptide antibiotics actinomycin X2 (AX2), actinomycin D, and actinomycin X0β. Among them, AX2 and actinomycin D showed strong anti-tuberculosis activity (Wang et al., 2017b). However, all three of them showed strong antitumor activity, especially AX2 (Chen et al., 2012b). Liu Jun found that AX2 could significantly reduce the proliferation rate and induce the apoptosis of prostate cancer cells by regulating the PTEN/PI3K/AKT/mTOR signaling pathway (Liu et al., 2016). Meanwhile, it was also found that AX2 decreased the expression level of miRNA144, and the direct target gene of miR-144 was subsequently identified as mTOR (Liu et al., 2016). However, miR-144 acts as a tumor suppressor gene to regulate cell proliferation, apoptosis and autophagy (Xiao et al., 2015; Wang et al., 2017c; Zhang et al., 2020b). In addition, it is well known that mTOR is a positive feedback regulator of the PI3K/Akt/mTOR pathway. Therefore, these data suggest that the antitumor effect of AX2 *via* modulation of the PTEN/PI3K/AKT/mTOR pathway may be somewhat attenuated by downregulation of miR-144.

#### 4.1.2 lncRNA-related marine-derived agents

In addition to miRNAs, several lncRNAs have also been implicated in the antitumor activity of marine-derived drugs, including the tumor suppressors LINC00261 (Ma et al., 2021), lncRNA-p21 (Yan et al., 2019), lncRNA LED and LOC285194 (Ramalingam et al., 2018) and the oncogenic lncRNA-Saf (Yan et al., 2019). As mentioned above, miR-29b- and miR-143-mediated signaling pathways play important regulatory roles in the activity of fucoidan against HCC (Yan et al., 2015; El-Far et al., 2021). It was further found that the lncRNA-mediated signaling pathway was also involved in the antitumor mechanism of fucoidan (Yan et al., 2019; Ma et al., 2021). LINC00261 was significantly upregulated by fucoidan in HCC cells and then acted as a tumor suppressor gene by regulating the miR-522-3p/secreted frizzled-related protein 2 (SFRP2) signaling pathway to inhibit cell proliferation and invasion (Ma et al., 2021). Similarly, lncRNA-p21 was also upregulated by fucoidan, which has been shown to inhibit the invasion and metastasis of HCC cells (Yan et al., 2019). In contrast, the expression of lncRNA-Saf was significantly downregulated by fucoidan in HCC cells, which has been shown to increase the anti-apoptotic ability of cells by affecting the alternative splicing of tumor necrosis factor (TNF) receptor superfamily member 6 (Fas) (Yan et al., 2005; Yan et al., 2019). These data suggest that fucoidan-mediated antitumor effects are closely related to the regulation of oncogene lncRNAs and tumor suppressor lncRNAs in tumors.

1-hydroxy-1-norresistomycin (HNM) is a secondary metabolite isolated from the marine actinomycete *Streptomyces* variabilis and has potent antimicrobial and antioxidant activity (Ramalingam and Rajaram, 2016). Recently, the antitumor effect of HNM was reported in human adenocarcinoma lung cancer cells. HNM can significantly inhibit cell proliferation, metastasis and invasion and promote cell apoptosis and cell cycle arrest. Mechanistically, p53-mediated transcriptional regulation of long noncoding RNAs (lncRNAs LED and LOC285194) was shown to be attributed to the repressive effect of HNM (Ramalingam et al., 2018). Among them, HNM-induced upregulation of the lncRNA LED can induce cell cycle arrest by targeting p21, while upregulation of LOC285194 can inhibit cell growth (Ramalingam et al., 2018). These findings also suggest the potential value of HNM as an anticancer drug by regulating tumor suppressor lncRNAs.

ASP-3 is a protein purified from marine *Arca subcrenata* and exhibits a strong inhibitory effect on the proliferation of HCC cells (Guo et al., 2019). Mechanistically, ASP-3 was found to effectively reduce vascular endothelial growth factor receptor (VEGFR) 2 phosphorylation (p-VEGFR2) in tumor cells and affect the vascular endothelial growth factor (VEGF) signaling pathway. Moreover, some tumor-related lncRNAs are regulated by ASP-3, including lncRNA ATG9B, which targets the VEGF signaling pathway (Guo et al., 2019). This suggests that ASP-3 can be used as an antitumor drug, and its molecular mechanism may be achieved by activating the ATG9B/VEGF signaling pathway to inhibit tumor angiogenesis-mediated cell invasion, which needs further study.

### 4.2 Regulation of ncRNA-related marine-derived agents in tumor drug sensitivity and resistance

#### 4.2.1 miRNA-related marine-derived agents

Cytarabine (AraC) serves as an essential cytotoxic agent in AML treatment, and AraC resistance is the main cause of treatment failure in patients (Dombret and Gardin, 2016). In the past two decades, many cytarabine resistance-related oncogenic or tumor-suppressive miRNAs have been identified in AML and other tumors. Generally, the expression of drug resistance-related tumor-suppressive miRNAs is depressed by AraC in tumor cells. For example, the expression levels of miR-143 and miR-181a were significantly reduced in Ara-C-treated AML cell lines and primary AML cells (Bai et al., 2012; Zhang et al., 2020c). Ectopic expression of miR-143 or miR-181a significantly reduced cell viability in Ara-C-treated AML cells, suggesting that these two miRNAs could enhance the sensitivity of AML cells to Ara-C (Bai et al., 2012; Zhang et al., 2020c). Mechanistically, miR-143 inhibited the induction of autophagy by targeting the autophagy-related genes (ATG)7 and ATG2B, thereby eliminating the inhibitory effect of autophagy in cytarabine-induced AML cell-dependent apoptosis and cytotoxicity (Zhang et al., 2020c). miR-181a was found to target Bcl-2 to enhance cytochrome C release, which in turn activates caspases 9/3 and ultimately initiates the apoptosis pathway (Bai et al., 2012). Moreover, overexpression of miR-12462, let-7a, miR-29a, or miR-30c was also found to sensitize AML cells to Ara-C *in vitro* and *in vivo* (Russ et al., 2011; Chen et al., 2013b; Jia et al., 2020). Furthermore, let-7a was proven to be involved in stromal-derived factor 1α (SDF-1α)/C-X-C chemokine receptor type (CXCR)4 axis-induced Ara-C tolerance through the SDF-1α/CXCR4/Yin Yang 1 (YY1)/let-7a/c-MYC/B-cell lymphoma extra-large (BCL-XL) pathway (Chen et al., 2013b). Downregulation of sodium/hydrogen exchanger 1 (NHE1), an isoform in the Na+/H+ exchanger family, was then found in miR-12462-overexpressing AML cells (Jia et al., 2020), while downregulation of ITGB3, c-MYC and BCL-XL was shown in let-7a-overexpressing AML cells (Chen et al., 2013b). In addition, low expression of miR-625-3p was found in ALL cell lines, and its overexpression significantly increased cell sensitivity to Ara-C-induced apoptosis (Jiang et al., 2018).

Similarly, miR-134 and miR-181b were significantly downregulated in multidrug-resistant leukemia cells and samples from patients with relapsed/refractory AML (Lu et al., 2014; Chen et al., 2018a). Overexpression of miR-134 or miR-181b can significantly increase the sensitivity of cells to Ara-C and enhance the ability of Ara-C to induce apoptosis (Lu et al., 2014; Chen et al., 2018a). miR-134 was then proven to inhibit the phosphorylation of eukaryotic initiation factor 4E (eIF4E) by targeting mitogen-activated protein kinase-interacting kinases (MNKs), leading to the downregulation of the antiapoptotic proteins MCL-1 and BCL2 (Chen et al., 2018a). Among them, Mcl-1, together with HMGB1, was also involved in the miR-181b-mediated enhancement of cellular sensitivity to Ara-C (Lu et al., 2014), which suggests the positive regulatory effect of miRNAs on Ara-C-induced apoptosis. In addition, miR-24-3p was found to be significantly more highly expressed in Ara-C-sensitive AML cell lines than in Ara-C-resistant cell lines (Bhise et al., 2019), indicating the role of miR-24-3p in the maintenance of cellular sensitivity to Ara-C. dCMP deaminase (DCTD), an enzyme involved in the metabolic inactivation of cytarabine expression, was suggested to possibly mediate the function of miR-24-3p (Bhise et al., 2019). These results suggest that Ara-C resistance-associated tumor suppressor miRNAs can inhibit the development of resistance by enhancing cellular chemosensitivity, inhibiting drug-induced autophagy, or maintaining drug cytotoxicity in tumor cells.

Unlike the tumor suppressor miRNAs associated with Ara-C resistance, the expression of oncogenic miRNAs associated with Ara-C resistance is induced by this drug in chemoresistant AML cell lines or patients, such as miR-15a-5p, miR-21-5p (Vandewalle et al., 2021), miR-126-5p (Shibayama et al., 2015), miR-10a (Vu et al., 2021), miR-331 (Butrym et al., 2015), miR-21 (Riccioni et al., 2015), and miR-335 (Yingchun et al., 2015). Moreover, it was found that overexpression of miR-15a-5p, miR-21-5p (Vandewalle et al., 2021), or miR-126-5 (Shibayama et al., 2015) significantly decreased cytarabine-induced apoptosis in AML cells, while inhibition of miR-10 (Vu et al., 2021), miR-21 (Li et al., 2010), or miR-32 (Gocek et al., 2011) could effectively inhibit cell viability and enhance the sensitivity of cells to Ara-C-induced apoptosis. Mechanistically, miR-15a-5p and miR-21-5p contribute to cytarabine resistance by targeting programmed cell death 4 (PDCD4), ADP-ribosylation factor 2 (ARL2), and B-cell translocation gene 2 (BTG2) (Vandewalle et al., 2021). miR-21 and miR-32 negatively regulate the expression levels of PDCD4- and Bcl-2-interacting mediator of cell death (Bim), respectively (Li et al., 2010; Gocek et al., 2011). The expression of the antiaging gene *Klotho* and the phosphorylation level of AKT were significantly decreased by miR-126-5p (Shibayama et al., 2015). miR-10a was proven to be a regulator of the p53 signaling network (Vu et al., 2021). Its inhibition could induce a synergistic antitumor effect between the MDM2 inhibitor Nutlin-3a and cytarabine through the activation of apoptosis in AML cells mediated by the p53 signaling network and the elimination of the inhibitory effect of autophagy on apoptosis (Vu et al., 2021). In addition, miR-34a-5p may also be an oncogenic miRNA that reduces the cytotoxic activity of cytarabine in AML cells by targeting and regulating deoxycytidine kinase (DCK), an enzyme involved in the activation of cytarabine (Bhise et al., 2019), a possible disease pathway that needs further study. Moreover, the regulatory role of miRNAs, such as miR-200, on cellular cytarabine tolerance is also seen in colon cancer. miR-200c expression was significantly upregulated in colon cancer patients, and its silencing in tumor cells induced apoptosis and inhibited cell survival, migration, and invasion. Moreover, silencing miR-200c sensitized cellular chemosensitivity to Ara-C *in vitro* and *in vivo* by modulating the expression of PTEN and the phosphorylation of p53 (Chen et al., 2014). These results suggest that the response of tumor cells to therapeutic cytotoxic agents can be increased by decreasing the expression of these cellular drug resistance oncogenic miRNAs.

Regarding trabectedin, myxoid liposarcoma (MLS) is the most sensitive type of soft tissue sarcoma to this drug, which may be correlated to the expression of the FUS-CHOP fusion gene generated by the chromosomal translocation t (12; 16) (q13; p11) (Grosso et al., 2007; Forni et al., 2009). Nonetheless, it has been observed in the clinic that some patients bearing MLS remain resistant to long-term treatment with trabectedin. Analysis of miRNA expression profiles in trabectedin-sensitive myxoid liposarcoma (MLS) cells and trabectedin-resistant cells revealed that 47 miRNAs were differentially expressed, including 24 overexpressed miRNAs and 23 underexpressed miRNAs (Uboldi et al., 2012). The target genes predicted by these differentially expressed miRNAs mainly existed in the immune response, the apoptosis regulatory system and the cell cycle. Several miRNAs have been shown to be associated with drug resistance, such as miR-130a, miR-19a, and miR-125a-5p (Huang et al., 2018; Huang et al., 2019; Zhang et al., 2020d), which suggests their value as indicators in tumor treatment, prognosis, or recurrence. Compared with patient-bearing MLS, resistance to trabectedin is more common in patients with non-small cell lung cancer (NSCLC) with a low response rate (20%–30%) for many chemotherapy drugs (Fossella, 2002; Rosell et al., 2002). miR-195 has been shown to be downregulated in NSCLC and acts as a tumor suppressor gene to inhibit the progression of tumor cells by regulating proliferation, apoptosis, and senescence (Arima et al., 2014; Yongchun et al., 2014; Liu et al., 2015). Furthermore, miR-195 was found to sensitize NSCLC to microtubule-targeting agents, especially trabectedin, *in vitro* and *in vivo*. Moreover, it was found that the facilitative effect of miR-195 on trabectedin was achieved by targeting checkpoint kinase 1 (CHEK1) (Yu et al., 2018b). Inhibition of miR-195 or overexpression of CHEK1 contributes to the resistance of NSCLC cells to trabectedin (Yu et al., 2018b). This suggests the potential value of trabectedin tolerance-related miRNAs as indicators of efficacy, prognosis or relapse in this drug therapy.

#### 4.2.2 lncRNA-related marine-derived agents

Similar to marine-derived agent-related miRNAs, lncRNAs associated with marine-derived agent resistance are also common in AML to Ara-C. However, these lncRNAs associated with Ara-C resistance mainly act as oncogenes to suppress cellular chemosensitivity and induce drug resistance, including lncRNA DANCR, GAS6-AS2, HOTAIRM1 and MALAT1. The lncRNA DANCR is highly expressed as an oncogene in many tumors, including AML (Thin et al., 2018; Zhang et al., 2021b). Interestingly, Ara-C further increased the expression level of DANCR in AML cells, and DANCR knockdown significantly reduced the resistance of AML cells to Ara-C. Further studies revealed that DANCR was able to promote autophagy in Ara-C-treated AML cells *via* the miR-20a-5p/ATG16L1 signaling pathway (Zhang et al., 2021b). This suggests that DANCR enhances the antagonism of autophagy against cytarabine-induced apoptosis by promoting autophagy, thereby inducing resistance to cytarabine in AML cells. LncRNA GAS6-AS2, a tumor-associated lncRNA first discovered in AML cells, is abnormally highly expressed and exhibits oncogenic effects in various tumors, including AML (Bester et al., 2018). The first oncogene function identified for GAS6-AS2 was involved in mediating Ara-C resistance in AML cells. GAS6-AS2 overexpression significantly promoted AML cell resistance to Ara-C-induced apoptosis *in vitro* and *in vivo*. Mechanistically, it was proven that GAS6-AS2 could increase the upregulation of growth arrest-specific gene 6 (GAS6) ligand and its receptor tyrosine kinase (AXL) to promote cellular survival and resistance (Bester et al., 2018). Similarly, HOTAIRM1 and MALAT1 have usually been shown to act as oncogenes that are highly expressed in AML and various other human tumors. Knockdown of both enhanced the sensitivity of AML to Ara-C, decreased cell viability and increased apoptosis (Chen et al., 2020; Goyal et al., 2021). HOTAIRM1 knockdown also suppressed glucose consumption and lactate production in AML cells (Chen et al., 2020). Subsequently, the role of HOTAIRM1 was confirmed to be related to the Wnt/β-catenin/platelet-type phosphofructokinase (PFKP) signaling pathway, while MALAT1 could target and regulate the tumor suppressor gene miR-96C (Chen et al., 2020; Goyal et al., 2021). In addition, TUG1 expression was elevated in refractory or relapsed acute myeloid leukemia (R/R AML) patients who received Ara-C combined with other drugs. It was further found that patients with high TUG1 expression had a lower overall response rate (ORR), lower complete response (CR) value, and shorter overall survival (OS) (Luo et al., 2018). This suggests that TUG1 is a potential biomarker for poor prognosis in R/R AML patients treated with Ara-C, but the relationship between TUG1 and tumor cell resistance to Ara-C requires further study.

## 5 The antitumor effects of marine food-derived ncRNAs

**FIGURE 5:**
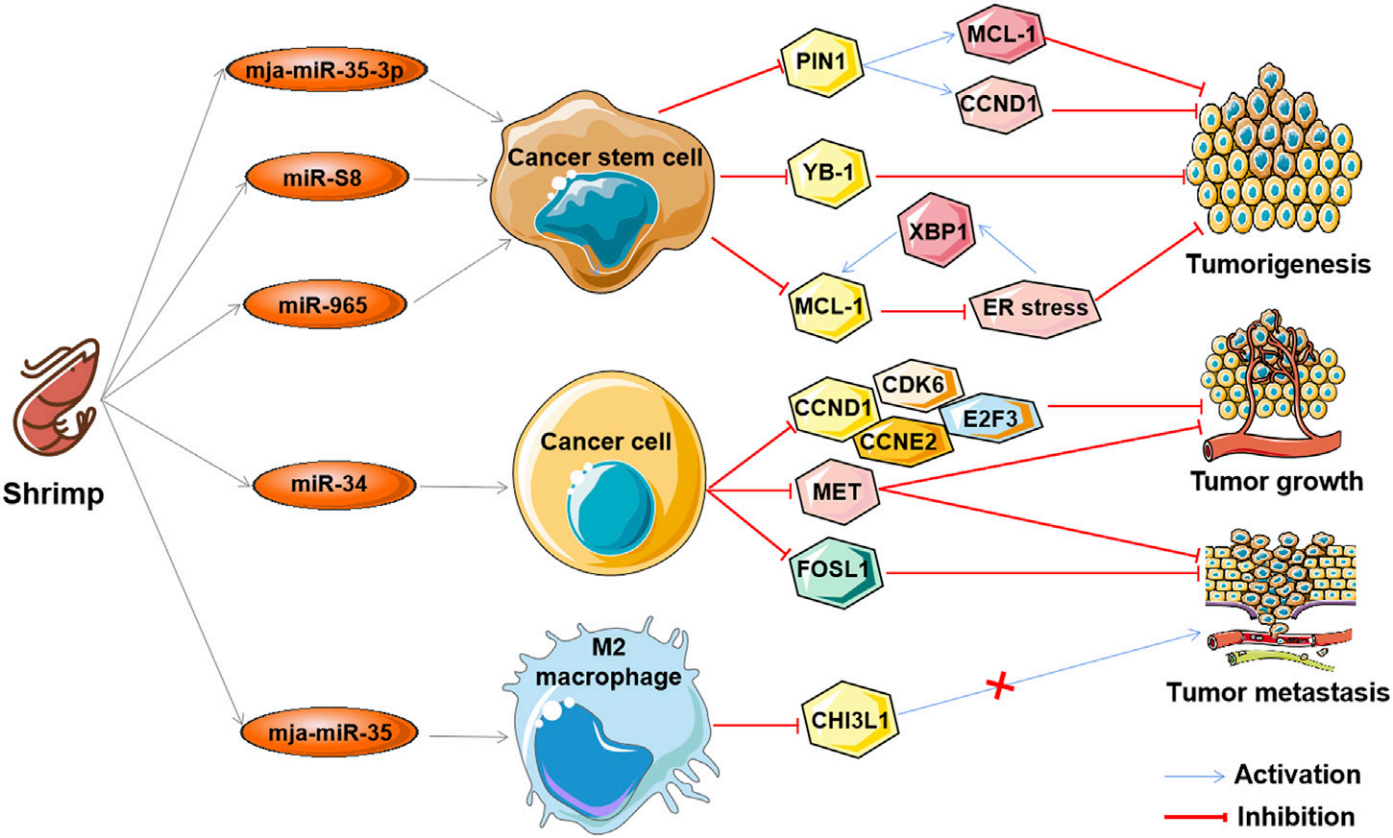
The antitumor effects of marine shrimp-derived microRNAs. microRNAs (miRNAs) derived from marine shrimp (*Marsupenaeus japonicus*) exhibit antitumor ability by regulating cross-species gene expression to inhibit human tumorigenesis, tumor growth, and tumor metastasis.

The aforementioned ncRNAs in tumors play important regulatory roles as oncogenes or tumor suppressors in the antitumor effects of marine-derived drugs in different clinical research stages, as shown in Table 3. Interestingly, recent studies have found that ncRNA in marine food can play an anti-tumor role by mediating gene expression across species, which further confirms the anti-tumor strategy of marine ncRNA targeting (shown in Figure 5). These marine food-related ncRNAs were mainly from shrimp fed by miRNA-expressing bacteria, including mja-miR-35-3p, miR-S8, miR-965, miR-34, and mja-miR-35 (Table 4). This suggests that the active ingredients of marine-derived antitumor drugs are not limited to polyphenols, polysaccharides, alkaloids, or peptides but may also be ncRNAs.

**TABLE 3:** ncRNA-associated marine-derived anti-tumor agents.

Agent name	Source	Late-stage of clinical development	Related ncRNA types	ncRNA roles in tumor	ncRNA-related tumor types
Eribulin	*Halichondria okadai*	Approved	miRNA	Suppressor	Breast cancer
Maritoclax	*Streptomyces*	Approved	miRNA	Suppressor	Renal carcinoma
Trabectedin	*Ecteinascidia turbinata*	Approved	miRNA	Oncogene or suppressor	Biliary tract cancer; Myxoid liposarcoma; Lung cancer
Cytarabine	*Cryptotethia crypta*	Approved	miRNA; lncRNA	Oncogene or suppressor	Acute myeloid leukemia; Colon cancer
Spirulina	*Cyanobacteria*	*In vivo*	miRNA	Suppressor	Glioma
ASP-3	*Arca subcrenata*	*In vivo*	lncRNA	Oncogene	Hepatocellular carcinoma
Fucoidan	Brown seaweed	*In vivo*	miRNA; lncRNA	Suppressor	Hepatocellular carcinoma
SZ-685C	Angrove endophytic fungus	*In vitro*	miRNA	Oncogene	Pituitary tumor
Bostrycin	*Alternaria eichhorniae*	*In vitro*	miRNA	Suppressor	Lung cancer
1386A	Mangrove fungus	*In vitro*	miRNA	Suppressor	Breast cancer
Actinomycin X2	*Streptomyces*	*In vitro*	miRNA	Oncogene	Prostate cancer
1-hydroxy-1 -Norresistomycin	*Streptomyces*	*In vitro*	lncRNA	Suppressor	Lung cancer

**TABLE 4:** Marine food-derived miRNAs as anti-tumor agents.

miRNAs	Source	Related human cellular processes	Related human genes	Tumor cell types	References
mja-miR-35-3p	*Marsupenaeus japonicus*	Inhibition of cell proliferation, Induction of cell cycle arrest and apoptosis	PIN1, CCND1, MCL-1	Melanoma stem cells, breast cancer stem cells	Zhang and Zhang, (2019)
miR-S8	*Marsupenaeus japonicus*	Inhibition of cell stemness and tumorigenic ability	YB-1	Melanoma stem cells	Yang et al. (2017)
miR-965	*Marsupenaeus japonicus*	Inhibition of cell stemness and tumorigenic ability	MCL-1, XBP1, ER stress	Melanoma stem cells	Wu et al. (2020)
miR-34	*Marsupenaeus japonicus*	Inhibition of cell tumorigenesis, tumor growth, and metastasis	CCND1, CDK6, MET, CCNE2, E2F3, FOSL1	Breast cancer cells	Cui et al., (2017); Cui et al., (2021)
mja-miR-35	*Marsupenaeus japonicus*	Inhibition of cell tumorigenesis, tumor growth, and metastasis	M2 macrophage, CHI3L1	Breast cancer cells	Chen et al. (2018b)

mja-miR-35-3p is a microRNA derived from marine shrimp (*Marsupenaeus japonicus*), and its expression can be significantly upregulated by white spot syndrome virus (WSSV), which in turn plays an important role in the antiviral infection of shrimp (Huang et al., 2012). Overexpression of mja-miR-35-3p in human melanoma and breast cancer stem cells inhibited cell proliferation, arrested the cell cycle, and induced cellular apoptosis (Zhang and Zhang, 2019). Furthermore, it was found that mja-miR-35-3p could target and decrease the upregulated peptidylprolyl cis/trans isomerase NIMA-interacting 1 (PIN1) in human melanoma and breast cancer stem cells, thereby leading to the elimination of the stemness of tumor stem cells (Zhang and Zhang, 2019). In addition, the anti-human tumor effect of mja-miR-35 on the tumorigenic ability of melanoma stem cells *in vivo* was confirmed. Similarly, the shrimp microRNAs miR-S8 and miR-965 were also found to inhibit the stemness of human melanoma stem cells *in vitro* and *in vivo* (Yang et al., 2017; Wu et al., 2020). Mechanistic studies showed that miR-S8 targeted human Y-box binding protein 1 (YB-1) and inhibited its expression, thereby mediating the reduction in stem cell tumorigenicity (Yang et al., 2017). miR-965 suppresses the expression of human myeloid cell leukemia sequence 1 (MCL-1) and disrupts the balance of the MCL-1-endoplasmic reticulum (ER) stress-human X-box-binding protein 1 (XBP1) feedback loop by interacting with the human Ago2 protein (Wu et al., 2020). These results suggest that shrimp microRNAs can inhibit the stemness of human tumor stem cells by regulating human tumor-related genes in a cross-species manner.

Similar to mja-miR-35, shrimp miR-34 and mja-miR-35 are two other WSSV-associated miRNAs whose expression can be upregulated by the virus, which then suppress the virus by targeting the viral genes wsv330 and wsv359 or wsv140, wsv279, wsv309, and wsv361, respectively (Cui et al., 2017; Chen et al., 2018b; Cui et al., 2021). Overexpression of shrimp miR-34 or mja-miR-35 in human breast cancer cells significantly suppressed cell metastasis *in vitro* and *in vivo* (Cui et al., 2017; Chen et al., 2018b). The molecular mechanism of shrimp miR-34 against human tumors was shown to be related to shrimp miR-34-mediated expression of multiple human tumor-related genes, including cyclin D1 (CCND1), cyclin dependent kinase 6 (CDK6), cyclin E2 (CCNE2), E2F transcription factor 3 (E2F3) FOS-like antigen-1 (FOSL1), and hepatocyte growth factor receptor (MET) (Cui et al., 2017), while the tumor suppressor function of mja-miR-35 is achieved by targeting M2 macrophages and human chitinase-3-like protein 1 (CHI3L1) (Chen et al., 2018b). Interestingly, elevated levels of mature miR-34 or mja-miR-35 expression were also found in shrimp fed bacteria overexpressing miRNAs (bacteria-miR-34 or bacteria-mja-miR-35), even in cooked shrimp (Chen et al., 2018b; Cui et al., 2021). Moreover, food (bacteria-miR-34 or bacteria-mja-miR-35)-derived shrimp miR-34 or mja-miR-35 still contribute to the antiviral immune response of shrimp (Chen et al., 2018b). Further studies found that transfection of human breast cancer cells with shrimp miR-34 or mja-miR-35 extracted from the muscle of bacteria-miRNA-fed shrimp could significantly inhibit cell growth and metastasis (Chen et al., 2018b; Cui et al., 2021). In addition, the antitumor effects of these two shrimp miRNAs were also demonstrated in human breast cancer xenograft model mice that were fed the cooked muscle of shrimp fed with bacteria-miRNAs (Chen et al., 2018b; Cui et al., 2021). These results demonstrate that xenogeneic miRNAs can inhibit not only human tumorigenesis but also tumor growth and progression, suggesting their potential as antitumor drugs. Furthermore, these results also suggest that miRNAs in food may be an effective strategy for the simultaneous control of viral diseases in food animals and human cancers.

## 6 Conclusion and discussion

ncRNAs are critical regulators of gene expression at multiple biological levels and are involved in the pathophysiology of various diseases, especially tumors. Substantial evidence indicates the potential of ncRNAs as novel biomarkers for tumor diagnosis and prognosis and even drug targets. In addition, as mentioned above, ncRNAs have recently been shown to play important roles in the antitumor mechanisms of marine-derived agents as oncogenes or tumor suppressors. This provides a new understanding of the antitumor mechanism of clinical (including cytarabine, fucoidan, and trabectedin) or potential marine-derived agents (other compounds or derivative derivatives mentioned in the text), which can promote their more rational clinical application or clinical translation. This also suggests that ncRNAs from marine life might be an important source for discovering human antitumor agents by mediating cross-species gene expression. Moreover, this further supports the potential of marine-derived agent-associated ncRNAs as biomarkers for tumor therapy, prognosis and recurrence, especially miRNAs. For example, Natsuko showed that serum miRNAs could serve as biomarkers of response to eribulin therapy in patients with metastatic breast cancer (Satomi-Tsushita et al., 2019). Of the 147 patients, 52 developed at least one new distant metastasis after treatment, and the remaining 95 did not develop new distant metastases. A combination of 8 serum miRNAs was predicted to be related to new distant metastases in patients after eribulin treatment, including miR-4483, miR-8089, miR-4755-3p, miR-296-3p, miR-575, miR-4710, miR-5698, and miR-3160-5p. Among them, miR-8089 and miR-5698 were also significantly associated with the overall survival of patients treated with eribulin (Satomi-Tsushita et al., 2019). This study demonstrates that serum miRNA profiles can serve as biomarkers of responsiveness to eribulin and predict new distant metastases and prognostic development in metastatic breast cancer. In a phase II and phase III clinical trial investigating the antitumor activity of eribulin in patients with metastatic STS, 26 microRNA biomarkers in tumor samples were associated with sensitive or resistant patients (Wiemer et al., 2017). This suggests that the expression levels of these specific microRNAs in STS tissue samples can be used as predictors of patient response to eribulin. In addition, Li found in the serum and bone marrow of 204 Ara-C-treated adult AML patients that the expression level of miR-335 was significantly higher in patients who achieved complete remission than in those who did not (Yingchun et al., 2015). Interestingly, high bone marrow miR-335 levels, but not serum miR-335 levels, were significantly associated with poorer treatment response and predicted poorer prognosis (Yingchun et al., 2015). This suggests that bone marrow miR-335 levels can be used as a biomarker for predicting chemotherapy response and prognosis in Ara-C-treated adult AML patients.

However, the antitumor activity of some marine-derived substances that may have antitumor effects and their molecular mechanisms related to ncRNAs need to be further studied, such as sponge FAU and sponge FAU-derived SNORA62. Sponge FAU is a gene/protein isolated from the marine sponge *Suberites domuncula* (Perina et al., 2015)*.* It has been shown to promote apoptosis of HEK293T cells, which suggests that sponge FAU may have the same potential for cancer treatment as human FAU (Pickard et al., 2011; Perina et al., 2015)*.* Moreover, the snoRNA SNORA62 was identified in the sponge FAU gene, which is the sponge ortholog of human SNORA62 (E2) (Perina et al., 2015). Human SNORA62 is the most upregulated gene in diallyl sulfide (DAS)-induced apoptosis in HeLa cells (Moodley and Weiss, 2013). This finding indicates that FAU-derived SNORA62 may be a tumor-related snoRNA involved in the regulation of the apoptotic system*.* In addition, research on the role of other types of ncRNAs in marine-derived antitumor drugs has not been reported and is worthy of study. After all, they also play very important regulatory functions in the occurrence and development of tumors, such as circRNAs. Moreover.

Overall, the available data on ncRNAs as mediators, inhibitors, or drug sources support their indispensable roles in marine-derived antitumor agents by regulating tumor cell tumor cell proliferation, apoptosis, the cell cycle, autophagy, drug sensitivity, or resistance. Therefore, upregulation of drug-related tumor suppressor ncRNAs or elimination of drug-related oncogenic ncRNAs in tumor cells is one of the important options for promising biomarkers for diagnosis, prognosis, or targets for cancer treatment. However, it should be noted that, in most cases, ncRNAs are not the direct targets of these marine antitumor agents. The effect of these drugs on ncRNAs is most likely an indirect or incidental result, which may only contribute a small part of the anti-tumor activity. In addition, we also found that the correlation between ncRNA-related marine-derived agents and tumor drug sensitivity and drug resistance was mainly found in AML, which may be related to the complexity of the recurrence and treatment of hematological tumors, and may also indicate the cancer specificity of marine-derived agents, which all need to be further studied. Therefore, more mechanism studies are needed to clarify the role of ncRNA in the anti-tumor of these agents, including the identification of suitable ncRNA detection methods, the screening of ncRNA biomarkers, and the development of drug-specific ncRNA modulators or adjuvants. Moreover, the research mechanism of some compounds (or foods) mentioned in this review is still limited to the laboratory stage, even only at the level of cell experiment, which still needs to be fully tested *in vivo* and follow-up clinical trials. With the development of marine biology, marine biochemistry, and marine pharmacy based on high-throughput sequencing and screening techniques, an increasing number of early-stage antitumor drug candidates in preclinical research will be translated into clinical treatment, new marine-derived substances are being used in the development of antitumor drugs, and new marine-derived antitumor drug-related ncRNAs will be identified. Additionally, the efficacy and safety of ncRNA-related marine-derived antitumor drug or food in the cancer treatment will gradually become clear.

## Glossary

AKT: protein kinase B
AML: acute myeloid leukemia
AraC: cytarabine
BCL-2: B-cell lymphoma-2
BCL-XL: B-cell lymphoma-extra large
BTC: biliary tract cancer
CART: Charge-altering releasable transporter
ceRNA: competitive endogenous RNA
circRNA: circular RNA
ER: endoplasmic reticulum
EV: extracellular vesicle
HCC: hepatocellular carcinoma
K-RAS: Kirsten rat sarcoma viral oncogene homolog
lncRNA: long noncoding RNA
MCL-1: myeloid cell leukemia sequence 1
miRNA: microRNA
mRNA: messenger RNA
ncRNA: noncoding RNA
mTOR: mammalian target of rapamycin
NF-κB: nuclear transcription factor-κB
NK: natural killer
NP: nanoparticle
PDCD4: programmed cell death 4
piRNA: piwi-interacting RNA
PI3K: phosphatidyl inositol 3-kinase
PTEN: phosphatase and tensin homolog deleted from chromosome 10
RNAi: RNA interference
snoRNA: small nucleolar RNA
STS: soft tissue sarcoma
TRAIL: tumor necrosis factor related apoptosis inducing ligand
tsRNA: transfer RNA-derived small RNA
VEGF: vascular endothelial growth factor
Wnt: wingless-type MMTV integration site family
